# Inhibition of c-MYC-miRNA 19 Pathway Sensitized CML K562 Cells to Etoposide via NHE1 Upregulation

**DOI:** 10.1155/2022/9306614

**Published:** 2022-07-23

**Authors:** Shannan Cao, Qingqing Xiong, Bowen Ding, Xu Wang, Feng Wei, Qian Sun, Fan Yang, Jing Luo, Guoqiang Chang, Suxin Li, Jian Wang

**Affiliations:** ^1^Clinical Laboratory, Yantai Affiliated Hospital of Binzhou Medical University, Yantai 264000, China; ^2^Department of Hepatobiliary Cancer, Liver Cancer Center, Tianjin Medical University Cancer Institute & Hospital, National Clinical Research Center for Cancer, Key Laboratory of Cancer Prevention and Therapy, Tianjin's Clinical Research Center for Cancer, Tianjin 300060, China; ^3^Department of Breast Reconstruction, Key Laboratory of Breast Cancer Prevention and Therapy, Tianjin Medical University, Ministry of Education, Tianjin Medical University Cancer Institute & Hospital, Tianjin 300060, China; ^4^Department of Pharmacology, Harold C. Simmons Comprehensive Cancer Center, University of Texas Southwestern Medical Center, 5323 Harry Hines Blvd, Dallas, Texas 75390, USA; ^5^Department of Immunology, Department of Biotherapy, National Clinical Research Center for Cancer, Key Laboratory of Cancer Prevention and Therapy, Tianjin's Clinical Research Center for Cancer, Key Laboratory of Cancer Immunology and Biotherapy, Tianjin Medical University Cancer Institute & Hospital, Tianjin 300060, China; ^6^Department of Neurology, Tianjin Neurological Institute, Tianjin Medical University General Hospital, Tianjin 300052, China; ^7^Department of Pharmaceutics, China Pharmaceutical University, 24 Tongjiaxiang, Nanjing 210009, China

## Abstract

As a previously discovered target of DNA damage, Na^+^/H^+^ exchanger 1 (NHE1) plays a role in regulation of intracellular pH (pH_i_) through the extrusion of intracellular proton (H^+^) in exchange for extracellular sodium (Na^+^). Its abnormal expression and dysfunction have been reported in solid tumor and hematopoietic malignancies. Here, we reported that suppression of NHE1 in BCR-ABL^+^ hematopoietic malignancies' K562 cells treated with Etoposide was manipulated by miR-19 and c-MYC. Inhibition of miR-19 or c-MYC enhanced the expression of NHE1 and sensitized K562 cells to Etoposide *in vitro*. The *in vivo* nude mouse transplantation model was also performed to confirm the enhanced sensitivity of K562 cells to Etoposide by inhibiting the miR-19 or c-MYC pathway. TCGA analysis conferred a negative correlation between miR-19 level and leukemia patients' survival. Thus, our results provided a potential management by which the c-MYC-miRNA 19 pathway might have a crucial impact on sensitizing K562 cells to Etoposide in the therapeutic approaches.

## 1. Introduction

As an important biological event, DNA damage has been deeply described. Chemotherapeutic regimens based on DNA damage have been developed and have been in the service of cancer patients. Despite some intrinsic chemoresistant malignancies, tumors even frequently relapse as chemoresistant malignancies also show dramatic initial responses to chemotherapy. Chemoresistance is thought to arise as a consequence of cell intrinsic genetic changes [1] including activation of detoxifying enzymes, upregulation of drug efflux pumps [2], apoptotic defects [3], and cell extrinsic factors such as cytokines and growth factors [4]. One outcome of chemoresistance is tumor relapse in the process of chemotherapy [5].

Relapse-associated factors need genetic change in different pathways. A pathway regulating the function of Bcl-xl has been described in several studies [6, 7]. In mouse thymocytes, DNA damage increases the activity of the amiloride-sensitive NHE1 to raise the intracellular pH (pHi), which in turn causes nonenzymatic deamidation of Bcl-xl [7]. Deamidation is due to its conversion of the amino acid asparagine to isoaspartic acid. Such an alteration reduces the ability of the antiapoptotic Bcl-xl protein to sequester and inhibit the BH3 only family of proapoptotic proteins, thereby promoting apoptosis [8]. The oncogenic kinase BCR-ABL conferred cells the capacity of antiapoptosis by affecting various pathways. BCR-ABL can inhibit the expression of NHE1, thus reserving the abundance of antiapoptotic Bcl-xl [9]. This may be a possible interpretation that chronic myeloid leukemia (CML) and other hematopoietic malignances resist apoptosis resulting from DNA damage.

NHE1, a highly conserved plasma membrane protein well established, regulate cell migration [10], proliferation [11], and death [9, 12] through the extrusion of intracellular proton (H^+^) in exchange for extracellular sodium (Na^+^) [13]. Increased NHE1 activity and/or expression has been shown in a variety of cancer types, including several breast cancer cell types [14], and has been proposed to be an early event in transformation especially via increased survival [15] and migratory/invasive properties [10]. Experimental evidences have linked NHE1 downregulation with inhibition of cell growth and enhanced apoptotic sensitivity [16, 17]. It seems that NHE1 play different roles between solid tumor and hematopoietic malignancies especially in BCR-ABL-positive myeloproliferative disorders. Solid tumor cells need NHE1 to maintain their malignancy and survival via pathways responsible for invadopodial ECM proteolysis, apoptosis, and intracellular redox regulation.

Additionally, previous studies did not elucidate precise tache responsible for the DNA damage-induced NHE1 expression.

In this paper, we reported that suppression of NHE1 in BCR-ABL^+^ hematopoietic malignancies' K562 cells treated with Etoposide was manipulated by miR-19. Inhibition of miR-19 or c-MYC could sensitize K562 cells to Etoposide. The nude mouse transplantation model was also performed to confirm the enhanced sensitivity of K562 cells to Etoposide by inhibiting the miR-19 or c-MYC pathway. TCGA analysis confers a negative correlation between miR-19 level and leukemia patient overall survival. Our results provide an insight to the c-MYC-miR-19-NHE1 pathway, which might have a crucial impact on triggering Etoposide-induced apoptosis.

## 2. Materials and Methods

### 2.1. Cell Lines, Cell Culture, Reagents, and Antibodies

The noninvasive human breast cancer cell line MCF-7 and hematopoietic malignant cell lines K562, HL60, and Jurkat were preserved by our laboratory. The highly invasive human breast cancer cell line MDA-MB-231 was a generous gift from Chong Qing Medical University. Cells were cultured in Dulbecco's modified Eagle's medium or RPMI 1640 medium (Gibco-BRL Life Technologies, Inc. Burlington, ON, CA) supplemented with 10% FBS (Hyclone, Logan, US), 100 *μ*g/mL streptomycin, and 100 U/mL penicillin, at 37°C in a 5% CO_2_ humidified incubator. We purchased the dual-excitation ratiometric pH indicator “BCECF-AM” from Thermo Fisher Scientific (US), the tyrosine kinase inhibitor “Imatinib” from Novartis (Switzerland), the cytotoxic chemotherapy drug “Etoposide,” the JAK2/STAT3 pathway inhibitor “AG490,” the c-Myc inhibitor “10058-F4,” and the selective NHE1 inhibitor “Cariporide” from Sigma-Aldrich (US). For Western blot analysis, we purchased anti-NHE1 antibody (Cat: sc-518041) and anti-*β*-actin antibody (Cat: sc-8432) from Santa Cruz Biotechnology (Santa Cruz, CA) and the Enhanced Chemiluminescence Reagent Plus (ECL) reagents from BD Biosciences (US).

### 2.2. RNA Isolation and Real-Time PCR

Total RNA was extracted using Trizol (Invitrogen, Grand Island, NY, US); 2 *μ*g RNA after treatment with DNase I (Invitrogen, Grand Island, NY, US) was reverse-transcribed using EasyScript kit (TransGen Biotech, BJ, China) following the manufacturer's instructions in a total volume of 20 *μ*L. Primers for real-time PCR were designed using Primer Premier software 5.0. Human *β*-actin primers used as an internal control were 5′-CCA CGA AAC TAC CTT CAA CTC C-3′ (forward) and 5′-ACT CGT CAT ACT CCT GCT TGC T-3′ (reverse). Human NHE1 primers were 5′-CCT GAC CTG GTT CAT CAA CA-3′ (forward) and 5′-TCA TGC CCT GCA CAA AGA CG-3′ (reverse). Real-Time PCR was performed with SYBR Green PCR kit (TransGen Biotech, BJ, China) on the ABI Prism 7500 Fast Sequence Detection System. Thermal cycling conditions were 95°C for 10 s, followed by 40 cycles of 5 s at 95°C, and 40 s at 60°C. PCR reactions were performed in a total volume of 20 *μ*L, containing 2 *μ*L of sample cDNA, 0.2 *μ*M of each primer, and the SYBR Green PCR mix following the manufacturer's instructions. Each test was amplified in three different wells in one experiment, repeated three times. Small RNA isolation, transcribing, and real-time PCR were performed by using miRcute miRNA kit (TIANGEN Biotech, BJ, China) according to the manufacturer's instructions.

### 2.3. Western Blotting

Proteins isolated from cell lines were resolved by 10% SDS-PAGE and transferred onto polyvinylidene difluoride membranes (Millipore, Bedford, MA). The membranes were blocked for 1 h with 5% skimmed milk in PBS and then incubated with primary antibodies and then horseradish peroxidase-conjugated secondary antibodies for 2 h and 1 h, respectively. Specific proteins were visualized with enhanced chemiluminescence detection reagent and determined by densitometric analysis with a Lynx video densitometer (Biological Vision).

### 2.4. Measurement of pH_i_

Cell suspensions in serum-free RPMI 1640 were washed and labeled with 10 *μ*M BCECF-AM for 15 min at 37°C. After loading, the chamber was flushed for 5 min with HEPES-buffered Ringer solution to remove any deesterified dye. The perfusion chamber was mounted on the stage of an inverted microscope (Leica), which was used in the epifluorescence mode with a ×40 oil immersion objective. BCECF was successively excited at 490/10 and 440/10 nm, and the resultant fluorescent signal was monitored at 535/10 nm using an intensified charge-coupled device camera and specialized computer software (MetaFluor). Between 10 and 20 cells were outlined and monitored during the course of the measurements. The results from each cell were averaged and taken for final analysis. Intensity ratio (490/440) data was converted into pH_i_ values using the high-K^+^/nigericin calibration technique. To this end, the cells were perfused at the end of each experiment for 5 min with standard high-K^+^/nigericin (10 *μ*g/mL) solution (pH 7.4). The intensity ratio data thus obtained was converted into pH values using the *r*_max_, *r*_min_, and p*K*a values previously generated from calibration experiments to obtain a standard nonlinear curve.

### 2.5. Construction of Reporter Plasmids

The DNA corresponding to -1360/+43 of the human SLC9A1 promoter region was PCR amplified from normal human genomic DNA using primers as 5′-GGC AGA TCT TTC CAG TGA TTC CAT TGT AC-3′ inserted BglII site (forward) and 5′-ACT GAA GCT TCC AGA ACT AAC CCT AGC CC-3′ inserted HindIII site (reverse). The construct was termed as pGL3-1360. Additional deletions were made and inserted into pGL3 basic vector (Promega). Its deletion reporters were generated as follows. A series of forward primers were used in combination with the same reverse primer in a PCR with pGL3-1360 as template. The forward primers, each of which bears a BglII site, were 5′-GCA TAG ATC TGT TGT GGG TTG AAT TGTG-3′ (pGL3-1259); 5′-GCT AGA TCT ATG AAG ATG GAC TAA TTA GG-3′ (pGL3-1200); 5′-CCG CAG GTC TTA AGA TGA GGT AATT ACG-3′ (pGL3-1156); 5′-GCA TAG ATC TCA CCT GAG GTC AGG AGT TC-3′ (pGL3-1029); 5′-GCT AGA TCT TGA ATC CAG GAG GCA GAG GT-3′ (pGL3-891); 5′-TGCG AGA TCT CTG ACAT AGTC ACT ATT AC-3′ (pGL3-399). 3′ untranslated region (UTR) of NHE1 was cloned into pGL3-control vector with XbaI site using primers as follows: 5′-TCT TTT CTA GAG GGG CAG TAA CAC CAG GGC-3′ (forward); 5′-TGG CTC TAG ACG TGG TTG TCG ATG TCA CC-3′ (reverse). The same region was cloned into psiCHECK2 Vector (Promega, Madison, WI) using primers as 5′-TTC TCT CGA GAGG GGC AGT AAC AC CAG-3′ (forward) and 5′-ATA AGC GGC CGC GTG GTT GTC GAT GTC-3′ (reverse). Primers 5′-GAT TGT TCA ATA AGA GGG CGC TCCTG-3′ (forward) and 5′-GCG CCC TCT TAT TGA ACA ATC ATA TAT AGG-3′ (reverse) were used to carry out mutation toward miR-19 targeted region in NHE1 3′ UTR. The shRNA sequence targeting TCA TTC CGT CAC TGA TCAT, GAT AGG TTT CCA TGT GATC, and CGA AGA GAT CCA CAC ACAG of NHE1 cDNA was designed and cloned into pSilencer U6 vector (Ambion Inc. US). All the plasmids were verified by direct sequencing at Invitrogen.

### 2.6. Cell Transfection and Luciferase Reporter Assay

Cells (1.00 × 10^6^ cells) were transfected with 800 ng of the indicated reporter plasmid together with 2.5 ng of the internal control plasmid pRL-TK using Lipofectamine 2000 (Invitrogen) according to the manufacturer's protocol. After transfection, the cells were treated with indicated inhibitors or DMSO for 24 h. Cells were then harvested and lysed. The luciferase activity was measured using the dual-luciferase reporter system (Promega) according to the manufacturer's instructions. The relative firefly luciferase activities were calculated by normalizing transfection efficiency. pECE vector was a gift of Shigeo Wakabayashi, Department of Molecular Physiology, National Cardiovascular Center Research Institute, Osaka, Japan. Obvious ectopic expression was achieved after 3 *μ*g pECE was transfected into MCF-7 cells using Lipofectamine 2000.800 ng shRNA vectors of NHE1 and 100 nM inhibitors of miR-19a and miR-19b (synthetized by GenePharma, Shanghai, China) were transfected using Lipofectamine 2000.

### 2.7. MTT Assay

Cells were seeded into 96-well culture plates at a density of 5 × 10^4^ cells/mL. Serial concentration of Imatinib or Etoposide and other inhibitors were added as indicated. After the drug treatment for indicated time, the medium was replaced with an equal volume of fresh medium containing 0.5 mg/mL MTT and incubated for 4 h at 37°C. Then, the medium was removed and 100 *μ*L DMSO was added and incubated for 10 min at room temperature. The cytotoxic effects of drugs were determined according to the OD values using a microplate reader at absorption wavelength of 576 nm. Data were background subtracted and normalized to control.

### 2.8. Apoptosis Assay and Flow Cytometry Analysis

For apoptosis analysis, cells were cultured at 5 × 10^4^ cells/mL in the presence of various concentrations of inhibitors or drugs as indicated. The percentage of apoptotic cells was evaluated using an Annexin V-PE-7AAD kit (BD Biosciences, NY, US) by LSRII cytometry system; GFP-positive cells were evaluated by FlowJo 10.8.1.

### 2.9. Biotin Pull-Down Assay

Biotin pull-down assay was carried out as described previously [18]. Briefly, nuclear extract was made from target cells. The extracts were incubated with biotin-labeled wild-type or SLC9A1 mutant double-stranded oligos. Streptavidin-sepharose beads (Sigma-Aldrich, US) were used to pull down biotin-associated complex.

### 2.10. Tumorigenicity Nude Mice

All animal experiments were performed in compliance with the guidelines of Laboratory Animal Care of National Institutes of Health for the care and use of laboratory animals and were approved by the institutional biomedical research ethics committee in the Laboratory Animal Center of Tianjin Medical University Cancer Institute and Hospital. We divided 4-week-old female BALB/C-nu/nu mice into certain groups according to our protocol. In subcutaneous models, 1 × 10^7^ K562 cells suspended in 0.2 mL PBS were injected into the right flank of each mouse at a single site. The tumors were monitored with a caliper after tumor inoculation. miR-19 inhibitor or c-MYC inhibitor 10058-F4 was intratumorally administrated when the tumor was observable. Etoposide was then intratumorally administrated one day after the treatments above. Tumor volume for each mouse was determined (in cubic millimeter) by measuring in two directions and calculated as tumor volume = length × (width)^2^/2. All mice were kept in aseptic cages and killed 7 weeks after inoculation by cervical dislocation after anaesthesia.

### 2.11. Statistical Analyses

All experiments were performed at least three times, and the results are expressed as mean ± standard error. Student's *t*-test was used to compare the data from each group. *P* < 0.05 was considered as statistically significant.

## 3. Results

### 3.1. Inhibition of NHE1 Reversed Etoposide-Induced Apoptosis of BCR-ABL^−^ Cell Lines Not in BCR-ABL^+^ Cell Line

**Figure 1:**
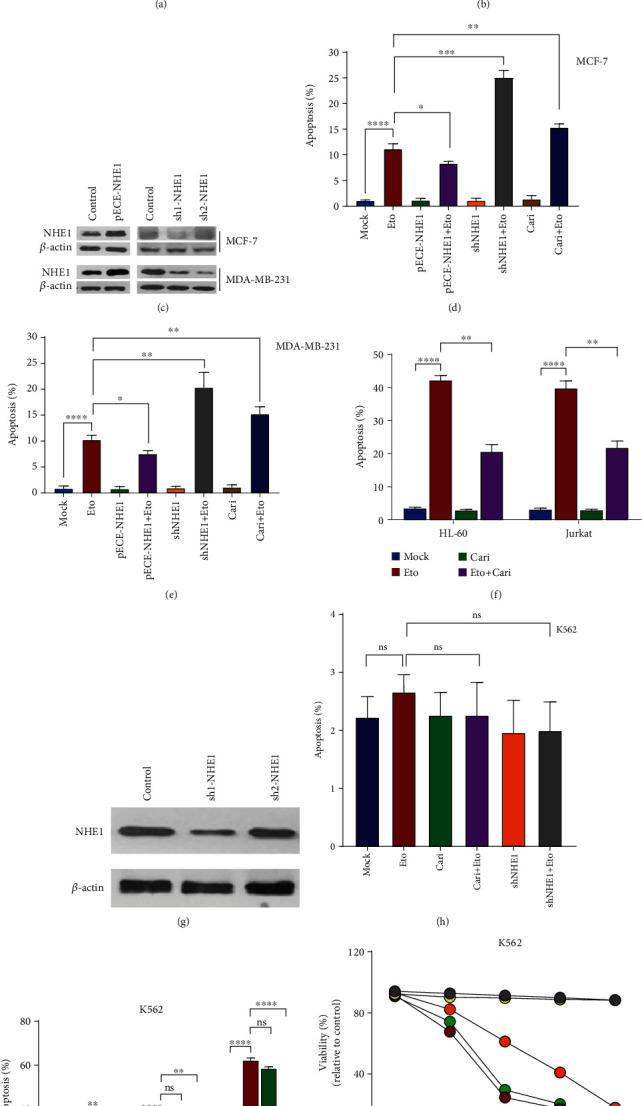
NHE1 affected the sensitivity of different cell lines to apoptosis. (a, b) MTT analysis of cells that were exposed to Cariporide (Cari) with or without Etoposide (Eto). OD values of untreated control cells were set as 100%. All other values refer to the untreated control. (c) Western blotting indicating the efficiency of pECE-NHE1 and two shRNA (sh1 and sh2) to NHE1 was carried out in MCF-7 and MDA-MB-231 cells. (d, e) Apoptosis of MCF-7 and MDA-MB-231cells transfected with indicated plasmids or treated with Cariporide was detected. Cells were transfected and then treated with Etoposide for 24 h. (f) Apoptosis of different hematopoietic cell lines exposed to Cariporide with or without Etoposide was shown. (g) Western blotting indicating the efficiency of two shRNA (sh1 and sh2) to NHE1 was carried out in K562 cells. (h) K562 cells were transfected with indicated shNHE1 plasmid or treated with Cariporide and then exposed to Etoposide. The apoptosis was shown. (i, j) K562 cells were transfected with indicated shNHE1 plasmid or treated with Cariporide and then exposed to Imatinib. The apoptosis and viability curves were shown. Data was shown as mean ± s.e.m. of triplicate assays. ^∗^*P* < 0.05. ^∗∗^*P* < 0.01. ^∗∗∗^*P* < 0.001. ^∗∗∗∗^*P* < 0.0001.

Firstly, we asked whether NHE1 plays different roles on Etoposide-mediated apoptosis in mammary cancer cell lines (MCF7 and MDA-MB-231), BCR-ABL^−^ hematopoietic cell lines (HL-60 and Jurkat), and BCR-ABL^+^ hematopoietic cell line (K562). In MCF-7 and MDA-MB-231 solid tumor cell lines treated with Etoposide, loss of viability was exacerbated by the additional presence of NHE1 specific inhibitor Cariporide along with time (Figures 1(a) and 1(b)). We then constructed NHE1-overexpressed and NHE1-knockdown MCF-7 and MDA-MB-231 cell lines, which was further confirmed by western blot (Figure 1(c)). Ectopic expression of NHE1 could partially reverse Etoposide-induced apoptosis in MCF-7 cells; accordingly, inhibition of NHE1 could further promote Etoposide-induced apoptosis (Figure 1(d)). The same phenomenon was also observed in MDA-MB-231 cells (Figure 1(e)). We then investigated the effect of NHE1 in hematopoietic cell lines. Interestingly, we reported the opposite effect of NHE1 in breast cancer cells compared with that in hematopoietic cells. Inhibition of NHE1 by Cariporide could reverse Etoposide-induced apoptosis in BCR-ABL^−^ HL-60 and Jurkat hematopoietic cell lines (Figure 1(f)), whereas the inhibition of NHE1 either by Cariporide or sh1-NHE1 showed no effect on Etoposide-induced apoptosis in BCR-ABL^+^ K562 hematopoietic cell line (Figures 1(g) and 1(h)). The results demonstrated that inhibition of NHE1 could not sensitize K562 cells to Etoposide. We then asked whether NHE1 may play roles in Imatinib-induced apoptosis in K562 cells since K562 has been reported sensitive to Imatinib. The results showed that inhibition of NHE1 either by Cariporide or shNHE1 could reverse Imatinib-induced apoptosis in K562 cells 24 h post Imatinib treatment (Figures 1(i) and 1(j)). Cariporide was shown to exert a transient effect to reverse apoptosis 24 h after Imatinib treatment, but it did not make K562 cells survive from cell death in long-time treatment. Based on the results found in hematopoietic cells, we hypothesized that Etoposide-induced apoptosis of BCR-ABL^−^ cells and Imatinib-induced apoptosis of BCR-ABL^+^ cells might be both through upregulation of NHE1. Whether manipulating NHE1 expression in BCR-ABL^+^ cells could sensitize cells to Etoposide is still unknown.

### 3.2. Etoposide-Induced Upregulation of NHE1 and pHi Value in BCR-ABL^−^ Cell Lines Not in BCR-ABL^+^ Cell Line

**Figure 2:**
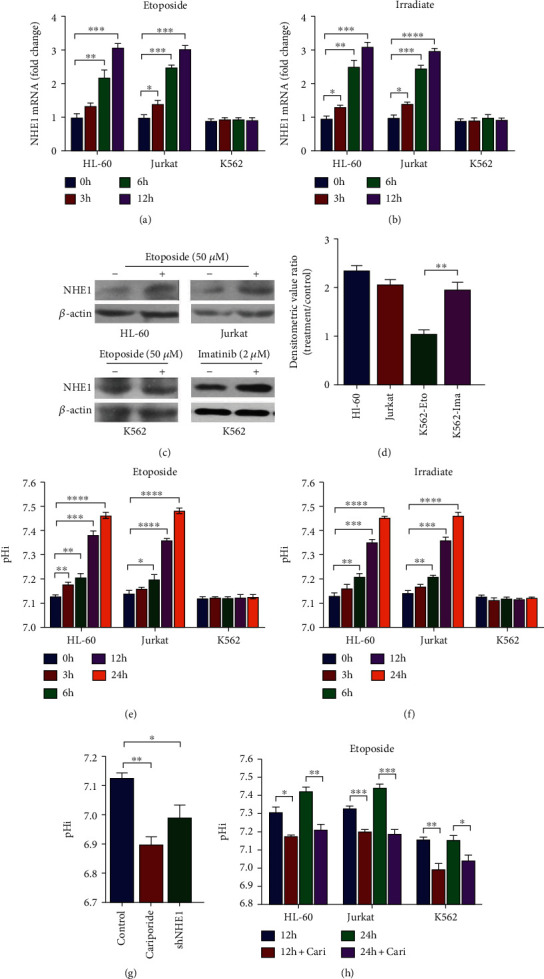
Etoposide altered NHE1 expression and pHi value among hematopoietic cell lines. (a) The mRNA expression level of NHE1 in HL-60, Jurkat, and K562 cells treated with Etoposide was detected. (b) The mRNA expression level of NHE1 in HL-60, Jurkat, and K562 cells treated with Irradiate was detected. (c, d) The protein expression level of NHE1 in HL-60 and Jurkat cells treated with Etoposide or in K562 cells treated with Imatinib was detected. Densitometric value was normalized by *β*-actin and showed the fold of treatment versus control. (e) The pHi in HL-60, Jurkat, and K562 cells treated with Etoposide was detected. (f) The pHi in HL-60, Jurkat, and K562 cells treated with Irradiate was detected. (g, h) The pHi in K562 cells with indicated treatments was detected. Data was shown as mean ± s.e.m. of triplicate assays. ^∗^*P* < 0.05. ^∗∗^*P* < 0.01. ^∗∗∗^*P* < 0.001. ^∗∗∗∗^*P* < 0.0001.

We have demonstrated that inhibition of NHE1 reversed DNA damage-induced apoptosis in HL-60 and Jurkat cells. We then detected the changes of NHE1 expression as well as pHi. As shown in Figures 2(a) and 2(b), expression of NHE1 increased 6 h posttreatment with Etoposide and Irradiate at the mRNA level. No obvious changes of NHE1 expression in K562 cells were observed. The detectable increased NHE1 expression 24 h post Etoposide treatment was further confirmed at the protein level in HL-60 and Jurkat cells (Figures 2(c) and 2(d)). As we have reported that upregulation of NHE1 might be important in Imatinib-induced apoptosis in K562 cells, we then detected NHE1 expression level in K562 cells treated with Imatinib. We know that upregulation could alter the pHi value and then we detected the pHi changes. We observed obvious increased pHi value in HL-60 and Jurkat cells, whereas no changes were found in K562 cells (Figures 2(e) and 2(f)). The specific inhibitor of NHE1 Cariporide could decrease pHi in all the three hematopoietic cell lines (Figures 2(g) and 2(h)). We have reported that inhibition with Cariporide could reverse Etoposide-induced apoptosis in HL-60 and Jurkat cells not in K562 cells. We then tried to investigate the potential mechanisms by which Etoposide suppressed NHE1 expression in K562 cells in the following studies.

### 3.3. Transcriptional Regulation Conferred to Suppressed NHE1 Expression in Etoposide-Treated K562 Cells

#### 3.3.1. Regulation of 5′ UTR of NHE1 in Etoposide-Treated K562 Cells

**Figure 3:**
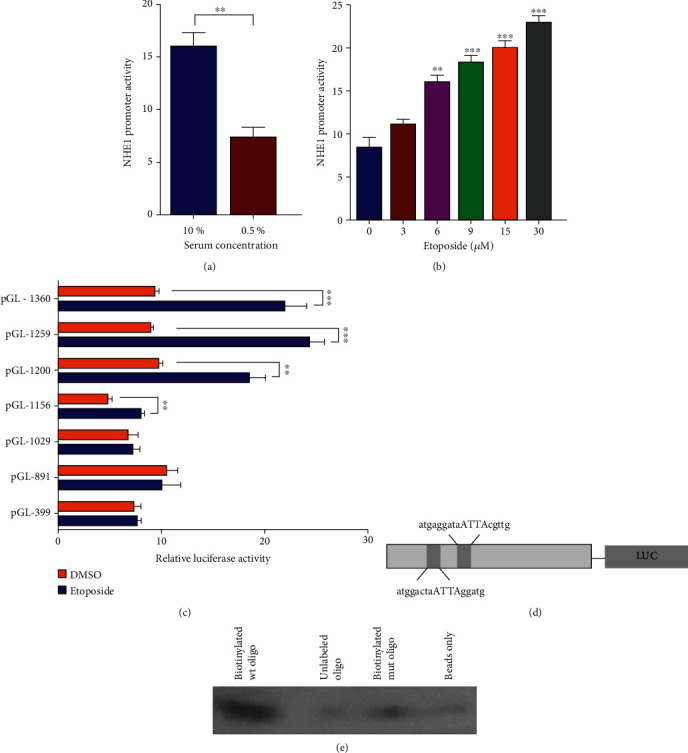
Characterization of NHE1 promoter activity responded to Etoposide. (a) SLC9A1 promoter activity responded to serum deprivation. (b) Luciferase activity in K562 cells transfected with PGL3-1360 and incubated with Etoposide at different final concentrations was detected. (c) Response of PGL3-1360 and its progressive deletion regions in K562 cells to Etoposide was clarified by relative luciferase activity. (d) A schematic diagram of the proximal SLC9A1 promoter indicating 2 potential OCT1 transcription factor binding sites. (e) Biotinylated oligo pull-down assay was performed to show the binding between OCT1 and target sequence. Data was shown as mean ± s.e.m. of triplicate assays. ^∗^*P* < 0.05. ^∗∗^*P* < 0.01. ^∗∗∗^*P* < 0.001. ^∗∗∗∗^*P* < 0.0001.

To detect the effect of DNA damage on NHE1 promoter activity, we used luciferase reporter plasmids in which 5′ flanking sequences of NHE1 promoter were constructed to the upstream of firefly luciferase coding sequences in the pGL3-basic vector. The plasmid pRL-TK driving moderate expression of Renilla luciferase was introduced as an internal control. Previous report showed that serum deprivation downregulated promoter activity of NHE1 [11]. Then, we detected the relative luciferase activity as positive control to see whether the system shows similar response in accordance with the existing report. As expected, the level of luciferase activity from cells grown in 0.5% serum was lower than that in the concentration of 10% serum (Figure 3(a)). Then, we employed the dual reporter system to examine how SLC9A1 (NHE1 gene) *cis*-elements respond to DNA damage. As shown in Figure 3(b), the transactivation of the SLC9A1 promoter induced by Etoposide was in a dose-dependent manner. The relative luciferase activity level was almost 3-folds relative to control in cells treated with Etoposide at 30 *μ*M. It suggests that the regulation of NHE1 expression could occur at the transcriptional level. To further determine the essential responsive elements for activation of SLC9A1 promoter activity in K562 cells, we generated serial luciferase reporter constructs with progressive deletion mutants from the entire SLC9A1 promoter to analyze the transactive effect of different regions. The relative luciferase activity of different constructs responding to Etoposide at 30 *μ*M in K562 cells is shown (Figure 3(c)). The constructs with 5′ flanking regions of 1360, 1259, 1200, and 1156 showed obvious response to Etoposide, whereas this activity was totally abrogated when the deletion achieved to -1029 relative to TSS. We inferred that the responsible element is located in the -1360~-1200 region. We then used Genomatix software, which implements identification of putative transcription factor-binding sites (Figure 3(d)). We identified four possible motifs in human SLC9A1 5′ fragments that were potentially responsive to OCT1, a well-studied transcription factor. In this region, many possible *cis*-elements predicted by different algorithm were found. Among these putative elements, we found OCT1 was reported as a member in the DNA damage-associated pathways. One of the four putative matrices which is -1148 relative to TSS in SLC9A1 promoter region is the same as the sequence AACGTAA, which was found in another pathway involved in epigenetic regulation by BRCA1 and OCT1 [18]. The biotin pull-down experiment demonstrated the binding between OCT1 and this element (Figure 3(e)). The results showed that the 5′ UTR regulation could be active to Etoposide-treated K562 cells. The suppression of NHE1 expression in Etoposide-treated K562 cells might be due to 3′ UTR regulation.

#### 3.3.2. Regulation of 3′ UTR of NHE1 in Etoposide-Treated K562 Cells

**Figure 4:**
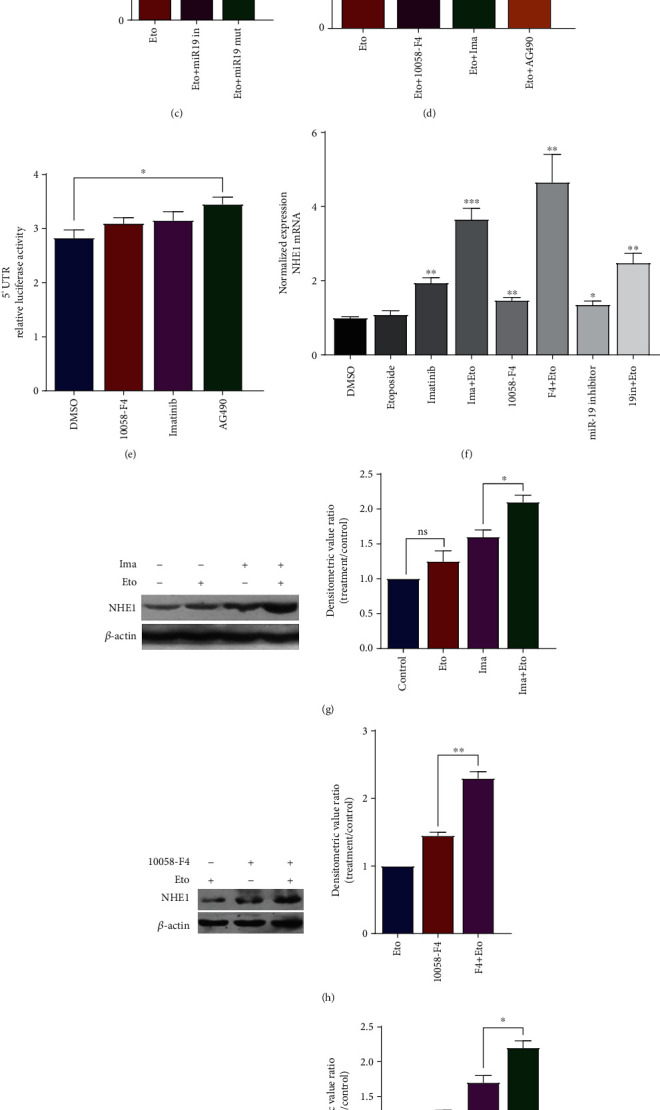
Identification of 3′ UTR of NHE1 mRNA as a possible target of miRNAs. (a) K562 cells transfected with pGL3-SLC9A1 showed lower relative luciferase activity than cells transfected with pGL3-control. This result was in according with what was found by introducing another luciferase report assay psiCHECK-2 vector constructed with the same sequence. (b) A schematic diagram of the putative seed regions in the 3′ UTR of NHE1. Highly conserved regions among vertebrates were shown in the diagram. By analyzing the sequence of 3′ UTR *in silico*, NHE1 was computationally predicted to be a target of multiple miRNAs, including hsa-miR-19. (c) The mutation of miR-19 binding site could increase activity of 3′ UTR, and the same trend was found by using miR-19 inhibitor. (d, e) Activity of SLC9A1 promoter and 3′ UTR responded to signaling inhibitors in K562 cells. (f) NHE1 expression at mRNA level was detected in K562 cells with indicated treatments. (g–i) NHE1 expression at protein level was detected in K562 cells with indicated treatments. (j) The pHi value in K562 cells with indicated treatments was detected. Data was shown as mean ± s.e.m. of triplicate assays. ^∗^*P* < 0.05. ^∗∗^*P* < 0.01. ^∗∗∗^*P* < 0.001. ^∗∗∗∗^*P* < 0.0001.

We have reported the intriguing finding that elements of 5′ UTR constructed in pGL3 vector could respond to Etoposide, whereas the expression of NHE1 was still suppressed in Etoposide-treated K562 cells. This finding implied that the downstream signal transmitter of DNA damage might persist in K562 cells. The controversial results between NHE1 expression and luciferase report assay of 5′ UTR may come from epigenetic regulation. We constructed luciferase report plasmid containing 3′ UTR of NHE1 mRNA and transfected into K562 cells. As shown in Figure 4(a), K562 cells transfected with pGL3-SLC9A1 showed lower relative luciferase activity than cells transfected with pGL3-control. This result was in accordance with what was found by introducing another luciferase report assay psiCHECK-2 vector constructed with the same sequence. It prompts that target elements in this region may play a role in regulating NHE1 expression. We then analyzed the sequence of 3′ UTR *in silico* and then found NHE1 was computationally predicted to be a target of multiple miRNAs, including hsa-miR-19 (Figure 4(b)). Among these possible miRNAs, hsa-miR-19 is a member of miR-17-92 cluster which was reported playing an important role in oncogene-induced senescence [19], TGF-*β* pathway regulation [20], and hypoxia-induced apoptosis [21]. Then, we investigated the potential role of miR-19 in NHE1 regulation in Etoposide-treated K562 cells. We first mutated the seed region according to the putative miRNA binding site in the reporter vector and detected the relative luciferase activity. The mutation of miR-19 binding site could increase activity of 3′ UTR, and the same trend was found by using miR-19 inhibitor (Figure 4(c)). Thus, we inferred that miR-19 may bind to 3′ UTR of NHE1 mRNA and consequently regulated NHE1 expression. In addition, we employed BCR-ABL pathway inhibitor Imatinib, c-MYC inhibitor 10058-F4, and JAK2 inhibitor AG490 to investigate if they can also regulate the activity of 3′ UTR of NHE1 mRNA. We found that Imatinib and 10058-F4 increased activity of 3′ UTR, whereas AG490 showed no obvious effect (Figure 4(d)). The activity of 5′ UTR in K562 cells treated with different inhibitors was also detected, and the result is shown in Figure 4(e).

#### 3.3.3. Manipulation of 3′ UTR Conferred to the Alteration of NHE1 Expression in Etoposide-Treated K562 Cells

We have showed that the manipulation of 3′ UTR of SLC9A1 might be responsible for suppressed NHE1 expression in Etoposide-treated K562 cells. Then, we asked whether the NHE1 expression could be upregulated by manipulating 3′ UTR activity. We detected the NHE1 mRNA expression as well as protein expression in Etoposide-treated K562 cells. The results showed that Imatinib, 10058-F4, and miR-19 inhibitor increased NHE1 expression both at mRNA and protein levels in K562 cells treated with Etoposide (Figures 4(f)–4(i)). Accordingly, we observed the increased pHi value in K562 cells treated with Etoposide (Figure 4(j)).

### 3.4. Inhibition of miR-19 and c-MYC Sensitized K562 Cells to Etoposide-Induced Apoptosis

**Figure 5:**
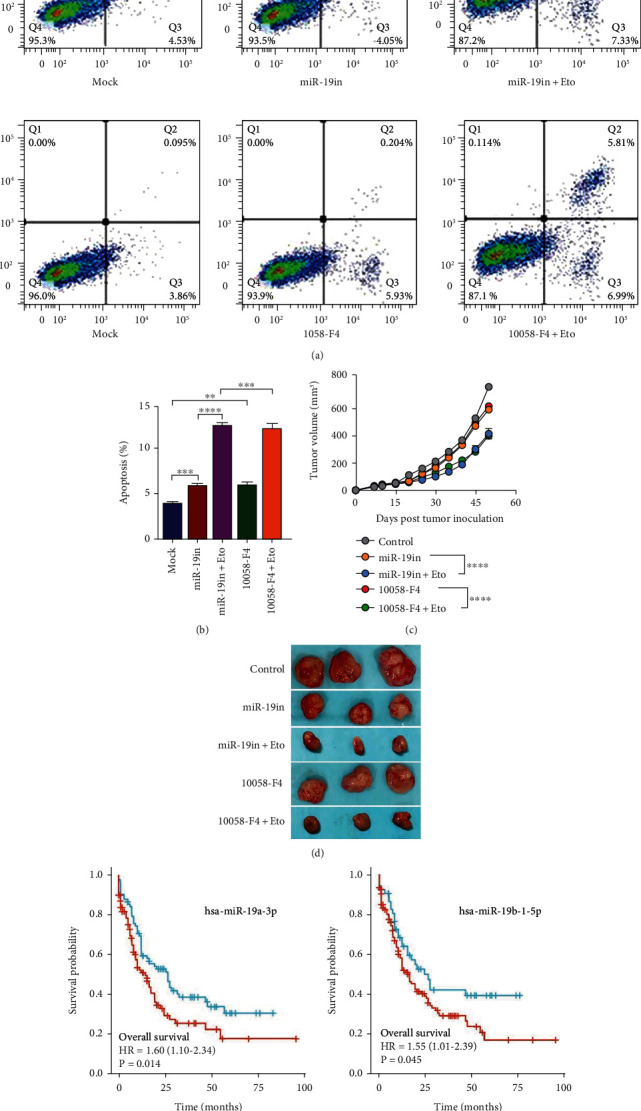
miR-19 and c-MYC affected apoptosis of K562 cells treated with Etoposide both *in vitro* and *in vivo*. (a, b) Inhibition of miR-19 or c-MYC showed synergistic effect with Etoposide treatment on K562 cell apoptosis. (c, d) Inhibition of miR-19 or c-MYC further inhibited tumorigenicity potential of K562 cells treated with Etoposide *in vivo*. Representative photographs of tumors taken at the time of killing were shown. The tumor volume for each mouse was determined (in cubic millimeter) by measuring in two directions and calculated as tumor volume = length × (width)^2^/2. The tumor growth curve was accordingly shown. (e, f) TCGA data showed the negative correlation between miR-19 expression level and overall survival of leukemia patients. The patients' overall survival curve was accordingly shown. Data was shown as mean ± s.e.m. of triplicate assays. ^∗^*P* < 0.05. ^∗∗^*P* < 0.01. ^∗∗∗^*P* < 0.001. ^∗∗∗∗^*P* < 0.0001.

Previous report showed that c-MYC is an important signal transmitter of BCR-ABL through its DNA binding and transcriptional activity [9]. It can promote miR-17-92 cluster expression including miR-19a and miR-19b which are corresponding to the same putative seed region in 3′ UTR of NHE1 mRNA. We employed 10058-F4 which can specifically inhibit the c-MYC-Max interaction and prevent transactivation of c-MYC target gene expression. We observed that the apoptosis of K562 cells was increased treated with miR-19 inhibitor and 10058-F4 (Figures 5(a) and 5(b)). We then further investigated the effect of 10058-F4 and miR-19 inhibitor on K562 tumor growth. In accordance with what we have reported *in vitro* results, miR-19 inhibition and c-MYC inhibition could reject tumor growth in nude mice (Figures 5(c) and 5(d)). Furthermore, we established the correlation between miR-19 expression level and leukemia cancer patients. We examined the cancer genome atlas (TCGA) to evaluate miR-19 from the current study in leukemia patients. Accordingly, low expression of miR-19 predicted better overall survival and high expression of miR-19 predicted poor clinical outcome (Figures 5(e) and 5(f)).

## 4. Discussion

NHE1 has been studied in a wide variety of tumor models and assigned an important role in the survival, proliferation, and invasive properties of tumor cells. A number of studies have shown that mRNA levels of NHE1 can be increased by a variety of external stimuli including serum [11], acidosis [22], PKC [23], and cell proliferation [24]. However, the mechanism by which the expression of NHE1 is induced after DNA damage has not been fully elucidated. We first found the change of NHE1 expression after DNA damage was at the mRNA level. Proximal region of SLC9A1 locus had a response to DNA damage uncovered by luciferase report assay in leukemia cell line K562. It prompted that DNA damage could activate transcriptional regulation for NHE1, although this regulation generated opposite results at mRNA level of NHE1. Then, we found the region responsible for the activation located in the -1360 to -1200 bp relative to the TSS. Although the same OCT1 matrix is putative and reported in another context after DNA damage [18], ChIP and knockdown experiments need to confirm if this binding is explicit and has a functional relevance *in vivo*. In this region, another putative transcription factor IKAROS possibly binds the region -1052 to -1044. Because IKAROS has been reported associated to apoptosis [25] and found aberrant in patients subjected to acute lymphoblastic leukemia [26], progressive experiments need to clarify this putative correlation.

Additionally, based on variability of the collection of binding sites, redundant transcription factors were computationally predicted. Thus, we cannot exclude other predicted transcription factors which were not reported to DNA damage and had a lower possibility to bind the given sequence. It needs to discover unknown binding proteins and other transcriptional regulator including miRNAs which contact with 5′ proximal NHE1 promoter.

When mentioned the inconformity between the luciferase activity and NHE1 mRNA expression in K562 cells, we doubt it might come from the epigenetic limitation to the locus, including modulation on CpG islands, histone modifications, distal enhancers and insulators, and other conformation regulation by nuclear organization. These events could not be modeled by the luciferase report assay based on transient transfection of plasmids. But our results prompt that the sensor and the transmitter for DNA damage in K562 cells work normally through the proximal promoters. In addition, as a possible epigenetic regulation, miRNA affected gene expression through mRNA degradation and translational suppression. We found the possible element responsible for the putative miR-19 binding sequence in the 3′ UTR of NHE1 mRNA. Consistent with this finding, inhibitor of miR-19 could sensitize K562 cells to Etoposide, although it seems not sufficient to induce remarkable apoptosis. Previous report showed that anti-c-MYC RNAi reduces miR-17-92 expression congruent with Imatinib treatment and inhibition of BCR-ABL gene expression by RNAi [27]. We reported that inhibitors of BCR-ABL, c-MYC and miR-19, can induce prominent apoptosis. We suspect that these miRNAs are downstream of this pathway which employs numerous other effectors simultaneously.

Previous report showed that NHE1 overexpression was sufficient to raise the intracellular pH, which in turn caused nonenzymatic deamidation of Bcl-xl in PBMCs and HSC from patients with CML [9]. But we did not observe similar change in K562 cells. We consider this diversity may come from progressive mutations brought into K562 cell line which represents terminal blast crises of CML. Thus, progressive insight of the miR-19–NHE1 pathway responsible to apoptosis and other biological function needs to be explored in a steady ectopic expression model *in vivo* to track the function along with the lineage commitment.

With regard to the solid cancer, NHE1 inhibition by Cariporide can sensitize MCF-7 and MDA-MB-231 cells to Etoposide-induced apoptosis. This is consistent with previous report in which NHE1 inhibition was implemented by another NHE inhibitor EIPA [17]. Ectopic expression of NHE1 could not increase the proliferation rate but could partially reverse the viability loss caused by Etoposide. We postulate that DNA damage can induce NHE1 expression and activate pathways enhancing the NHE1 activity. Different hormones and growth factors regulate the activity of NHE1 through the interaction with membrane receptors coupled with tyrosine kinases, G-proteins, calcineurin B homologous protein or integrins, resulting in a modulation of the cytoplasmic C-terminal regulatory domain. It affects Ras-ERK cascade, which includes different downstream effectors such as Raf-1, MEK1/2, and p42/44 MAPK [28]. In consideration of a variety of DNA-damaging agents that have been shown to activate MAP kinase in many different cell types, we speculated DNA damage may regulate the activity of NHE1 via these pathways. Meanwhile, MAP kinase pathways are involved in the regulation of the p53-independent induction of the GADD45 promoter via interaction with transcription factors that directly bind to OCT-1 motifs which were also located in the proximal promoter region [29]. Based on these clues and our results, we speculate that DNA damage regulates the expression and function of NHE1 possibly via MAP kinase and OCT-1-associated pathways.

Different roles of NHE1 have been found between hematopoietic cell lines and the mammary cancer cell lines. Ectopic NHE1 expression could sensitize K562 cells to Imatinib, while it would protect solid tumor cell lines from DNA damage-induced apoptosis. We hypothesize that different cell types have diverse balance between NHE1-regulated surviving and apoptosis. For solid tumors, contexts including serum deprivation [11] and cellular acidosis can increase NHE1 expression [22]. ROS [30] and HIF [31] are also reported to affect NHE1 function at diverse levels.

In addition to maintain the ion and proton balance, NHE1 was studied numerously in the metastasis. NHE1 colocate with CD44 and actin-binding protein especially ezrin, radixin, and moesin (ERM) family in pseudopodia and invadopodia [10]. NHE1-participating events result in sodium gradient and in turn drive the extrusion of protons, alkalinize intracellular pH, and acidify the extracellular pH (pH_e_). The low pH_e_ of the microenvironment might provide a proteolytically active environment that surrounds the tumor [32]. This optimizes the activity of the urokinase-type plasminogen activator, cathepsin systems and converts pro-MMPs to active MMPs mediating ECM degradation during tumor cell invasion [33]. Furthermore, previous report identified NHE1-associated immune complex contains the type II TGF-*β* receptor [34]. TGF-*β* induced by anticancer therapies can promote radiation-induced lung metastases of mammary tumors in mouse model requiring normal T*β*RII [35]. These clues suggest that NHE1 induced by DNA damage not only regulate apoptosis but also might promote tumor cells' metastasis to distal niche to search protection or to occupy new manor.

Collectively, our study showed that DNA damage could increase the transcriptional activity of SLC9A1 proximal 5′ promoter region containing responsible region which has putative OCT-1-binding matrices. DNA damage could induce NHE1 expression in BCR-ABL-negative HL-60, Jurkat, MCF-7, and MDA-MB-231 cell lines, while it could not affect NHE1 expression in BCR-ABL-positive leukemic cell line K562. Inhibition of NHE1 can decrease apoptosis in BCR-ABL-negative cell lines at DNA damage by Etoposide. In BCR-ABL-positive K562 cells, suppression of NHE1 expression change induced at DNA damage might result from miR-19 which is regulated by c-MYC. Inhibition of c-MYC, miR-19 sensitized K562 to Etoposide-induced apoptosis. Our results provide an insight to the miR-17-92-NHE1 pathway which possibly bypassed Imatinib resistance, but exact regulation and its functional relevance in hematopoiesis need further investigation.

## Data Availability

All data generated or analyzed during this study are included in this published article.

## References

[1] Zeller C., Hinzmann B., Seitz S. (2003). SASH1: a candidate tumor suppressor gene on chromosome 6q24.3 is downregulated in breast cancer. *Oncogene*.

[2] Zaboikin M., Srinivasakumar N., Schuening F. (2006). Gene therapy with drug resistance genes. *Cancer Gene Therapy*.

[3] Fulda S. (2012). Exploiting inhibitor of apoptosis proteins as therapeutic targets in hematological malignancies. *Leukemia*.

[4] Gilbert L. A., Hemann M. T. (2010). DNA damage-mediated induction of a chemoresistant niche. *Cell*.

[5] Visvader J. E., Lindeman G. J. (2008). Cancer stem cells in solid tumours: accumulating evidence and unresolved questions. *Nature Reviews. Cancer*.

[6] Zhao R., Oxley D., Smith T. S., Follows G. A., Green A. R., Alexander D. R. (2007). DNA damage-induced Bcl-xL deamidation is mediated by NHE-1 antiport regulated intracellular pH. *PLoS Biology*.

[7] Zhao R., Yang F. T., Alexander D. R. (2004). An oncogenic tyrosine kinase inhibits DNA repair and DNA-damage-induced Bcl- x_L_ deamidation in T cell transformation. *Cancer Cell*.

[8] Deverman B. E., Cook B. L., Manson S. R. (2002). Bcl-x_L_ deamidation is a critical switch in the regulation of the response to DNA damage. *Cell*.

[9] Zhao R., Follows G. A., Beer P. A. (2008). Inhibition of the Bcl-xL deamidation pathway in myeloproliferative disorders. *The New England Journal of Medicine*.

[10] Cardone R. A., Casavola V., Reshkin S. J. (2005). The role of disturbed pH dynamics and the Na^+^/H^+^ exchanger in metastasis. *Nature Reviews. Cancer*.

[11] Besson P., Fernandez-Rachubinski F., Yang W., Fliegel L. (1998). Regulation of Na+/H+ exchanger gene expression: mitogenic stimulation increases NHE1 promoter activity. *The American Journal of Physiology*.

[12] Grenier A. L., Abu-ihweij K., Zhang G. (2008). Apoptosis-induced alkalinization by the Na+/H+ exchanger isoform 1 is mediated through phosphorylation of amino acids Ser726 and Ser729. *American Journal of Physiology. Cell Physiology*.

[13] Orlowski J., Grinstein S. (1997). Na^+^/H^+^ exchangers of mammalian cells∗. *The Journal of Biological Chemistry*.

[14] Lin Y., Chang G., Wang J. (2011). NHE1 mediates MDA-MB-231 cells invasion through the regulation of MT1-MMP. *Experimental Cell Research*.

[15] Pang T., Wakabayashi S., Shigekawa M. (2002). Expression of calcineurin B homologous protein 2 protects serum deprivation- induced cell death by serum-independent activation of Na^+^/H^+^ exchanger∗. *The Journal of Biological Chemistry*.

[16] Lauritzen G., Jensen M. B. F., Boedtkjer E. (2010). NBCn1 and NHE1 expression and activity in DeltaNErbB2 receptor-expressing MCF-7 breast cancer cells: contributions to pHi regulation and chemotherapy resistance. *Experimental Cell Research*.

[17] Yang X., Wang D., Dong W., Song Z., Dou K. (2010). Inhibition of Na(+)/H(+) exchanger 1 by 5-(N-ethyl-N-isopropyl) amiloride reduces hypoxia-induced hepatocellular carcinoma invasion and motility. *Cancer Letters*.

[18] Shukla V., Coumoul X., Lahusen T. (2010). BRCA1 affects global DNA methylation through regulation of DNMT1. *Cell Research*.

[19] Hong L., Lai M., Chen M. (2010). The miR-17-92 cluster of microRNAs confers tumorigenicity by inhibiting oncogene-induced senescence. *Cancer Research*.

[20] Mestdagh P., Boström A. K., Impens F. (2010). The miR-17-92 microRNA cluster regulates multiple components of the TGF-*β* pathway in neuroblastoma. *Molecular Cell*.

[21] Taguchi A., Yanagisawa K., Tanaka M. (2008). Identification of hypoxia-inducible factor-1*α* as a novel target formiR-17-92microRNA cluster. *Cancer Research*.

[22] Krapf R., Pearce D., Lynch C. (1991). Expression of rat renal Na/H antiporter mRNA levels in response to respiratory and metabolic acidosis. *The Journal of Clinical Investigation*.

[23] Williams B., Howard R. L. (1994). Glucose-induced changes in Na+/H+ antiport activity and gene expression in cultured vascular smooth muscle cells. Role of protein kinase C. *The Journal of Clinical Investigation*.

[24] Rao G. N., Sardet C., Pouysségur J., Berk B. C. (1990). Differential regulation of Na+/H+ antiporter gene expression in vascular smooth muscle cells by hypertrophic and hyperplastic stimuli. *The Journal of Biological Chemistry*.

[25] He L. C., Xu H. Z., Gu Z. M. (2011). Ikaros is degraded by proteasome-dependent mechanism in the early phase of apoptosis induction. *Biochemical and Biophysical Research Communications*.

[26] Swafford A. D. E., Howson J. M., Davison L. J. (2011). An allele of IKZF1 (Ikaros) conferring susceptibility to childhood acute lymphoblastic leukemia protects against type 1 diabetes. *Diabetes*.

[27] Venturini L., Battmer K., Castoldi M. (2007). Expression of the miR-17-92 polycistron in chronic myeloid leukemia (CML) CD34+ cells. *Blood*.

[28] Baumgartner M., Patel H., Barber D. L. (2004). Na(+)/H(+) exchanger NHE1 as plasma membrane scaffold in the assembly of signaling complexes. *American Journal of Physiology. Cell Physiology*.

[29] Tong T., Fan W., Zhao H. (2001). Involvement of the MAP kinase pathways in induction of *GADD45* following UV radiation. *Experimental Cell Research*.

[30] Gaitanaki C., Mastri M., Aggeli I. K. S., Beis I. (2008). Differential roles of p38-MAPK and JNKs in mediating early protection or apoptosis in the hyperthermic perfused amphibian heart. *The Journal of Experimental Biology*.

[31] Shimoda L. A., Fallon M., Pisarcik S., Wang J., Semenza G. L. (2006). HIF-1 regulates hypoxic induction of NHE1 expression and alkalinization of intracellular pH in pulmonary arterial myocytes. *American Journal of Physiology. Lung Cellular and Molecular Physiology*.

[32] Izumi H., Torigoe T., Ishiguchi H. (2003). Cellular pH regulators: potentially promising molecular targets for cancer chemotherapy. *Cancer Treatment Reviews*.

[33] Kristine Glunde S. E. G., Solaiyappan M., Pathak A. P., Ichikawa Y., Bhujwalla Z. M. (2003). Extracellular acidification alters lysosomal trafficking in human breast cancer cells. *Neoplasia*.

[34] Karydis A., Jimenez-Vidal M., Denker S. P., Barber D. L. (2009). Mislocalized scaffolding by the Na-H exchanger NHE1 dominantly inhibits fibronectin production and TGF-beta activation. *Molecular Biology of the Cell*.

[35] Biswas S., Guix M., Rinehart C. (2007). Inhibition of TGF-beta with neutralizing antibodies prevents radiation-induced acceleration of metastatic cancer progression. *The Journal of Clinical Investigation*.

